# Production and Potential Application of Pyroligneous Acids from Rubberwood and Oil Palm Trunk as Wood Preservatives through Vacuum-Pressure Impregnation Treatment

**DOI:** 10.3390/polym14183863

**Published:** 2022-09-15

**Authors:** Chuan Li Lee, Kit Ling Chin, Pui San Khoo, Mohd Sahfani Hafizuddin, Paik San H’ng

**Affiliations:** 1 Institute of Tropical Forestry and Forest Product, Universiti Putra Malaysia, Serdang 43400 UPM, Selangor, Malaysia; 2 Centre for Advanced Composite Materials, Universiti Teknologi Malaysia, Johor Bahru 81310, Johor, Malaysia; 3 Faculty of Forestry and Environment, Universiti Putra Malaysia, Serdang 43400 UPM, Selangor, Malaysia

**Keywords:** pyroligneous acid, oil palm trunk, rubberwood, termite attack, mould fungi, wood decay fungi, vacuum-pressure impregnation, wood preservatives

## Abstract

The development of low-environmental-impact technologies for the elimination of biological damage is one of the vital goals of the wood protection industry. The possibility of utilizing pyroligneous acid as a wood preservative can be a great solution to extend the application of the currently fast-growing timber species, which has lower natural durability against biological damage. In this study, the effectiveness of pyroligneous acid as a wood preservative was evaluated by impregnating rubberwood with pyroligneous acid using vacuum-pressure treatment, and the treated woods were exposed to mould fungi, wood-decay fungi and termite attacks under laboratory conditions. Pyroligneous acids produced from rubberwood (RWPA) and oil palm trunk (OPTPA) at different pyrolysis temperatures were evaluated. To fully understand the effectiveness of pyroligneous acids as wood preservatives, different concentrations of pyroligneous acids were impregnated into rubberwood. Concentrations of 50% RWPA and 30% OPTPA were sufficient against mould and decay fungi on rubberwood. Rubberwood impregnated with pyroligneous acid acted as a slow-acting toxic bait to cause a high termite mortality rate due to toxic feeding and does not serve as a good repellent to prevent termites from feeding on the wood. In general, OPTPA has better biological durability compared to RWPA.

## 1. Introduction

For years, special attention has been given to biomass pyrolysis to increase the economic viability of this process while contributing to the reduction of the environmental pollution arising from waste accumulation and open field burning. One of the most important liquid products from wood pyrolysis is pyroligneous acid or wood vinegar, the aqueous portion obtained from the carbonization of wood and other lignocellulosic raw materials [1,2]. The chemical compounds in pyroligneous acid, which are derived from the pyrolysis process, depend on the biomass chemical composition—mainly, the three major components, i.e., cellulose, hemicellulose and lignin. The overall pyroligneous acid conversion level is related with interactions between the components and minus amounts of mineral matter that naturally present in whole biomass samples which catalyze numerous reactions taking place during pyrolysis and affect the final content of pyroligneous acid.

The main chemical compounds of pyroligneous acids include methanol, allylalcohol, acetic acid, acetone, acetaldehyde, methyl acetone, furan and furfural and formic, propionic and butyric acid [3]. Pyroligneous acids contains various acids (mainly, acetic acid) and phenolic compounds which have great antifungal properties [4,5]. Salim et al. [6] showed that 4-ethyl-2-methoxyphenol, 2, 6-dimethoxyphenol, guaiacol and cresol in pyroligneous acids showed good antifungal activity. Pyroligneous acid is currently used as a fungicide, fertilizer, soil enhancer, animal feed supplement and source of smoke flavoring for food [7,8,9]. However, further studies establishing the new functionality of pyroligneous acids as wood preservatives against biological degradation will increase the green value and the serviceability of one of the most prominent constructional materials, which is wood. 

The shortage in the supply of durable wood species has resulted in an increase in the use of plantation-grown timber species which have a lower natural durability, such as rubberwood [10]. In order to enhance the service life of these non-durable timber species, preservative treatment becomes necessary. Biological damage to wood and wood products is mainly caused by sap stain fungi, decay fungi and insects such as beetles and termites [11,12]. The development of low-environmental-impact technologies for the elimination of biological damage is one of the vital goals of the wood protection industry. Many chemicals are used to enhance the durability of wood and wood-derived products; public concerns remain over the use of these chemicals such as copper, chromium, arsenate, zinc, etc. Though these preservatives are useful in protecting wood from biodeterioration, environmental toxicity is also associated with the use of it. These chemicals are harmful to human health, our wildlife and the environment.

The possibility of utilizing pyroligneous acid as a wood preservative can be a great solution to extend the application of the currently fast-growing timber species, which has a lower natural durability against biological damage. Although much research has been conducted on the antimicrobial properties [13,14,15,16], none of the research has been carried out to study the effectiveness of pyroligneous acid as a wood preservative through vacuum-pressure impregnation treatment. The impregnation technique has been commercialized for over 70 years, and it remains the ideal technique for ensuring pressure-tight components in failsafe applications; the chemical penetrates deep into the wood with the pressure and vacuum [17,18,19]. Using this vacuum pressure impregnation method, the retention level of pyroligneous acid in wood can be improved. 

Therefore, in this study, the effectiveness of pyroligneous acid as a wood preservative was evaluated by impregnating rubberwood with pyroligneous acid using the vacuum pressure method and exposing the treated woods to mould fungi, wood-decay fungi and termite attacks under laboratory conditions. To fully understand the effectiveness of pyroligneous acids as wood preservatives, different dilution rates of pyroligneous acids were impregnated into the wood. Rubberwood was selected in this study as the testing substrate due to its low natural biological durability. The characteristics of pyroligneous acid from oil palm trunk and rubberwood sawdust produced from different pyrolysis temperatures were also evaluated.

## 2. Materials and Methods

### 2.1. Raw Materials Preparation

Rubberwood sawdust was collected from Langat Merah Sdn Bhd, located at Jenjarom, Kuala Langat, Selangor. Oil palm trunk (OPT) was collected from Business Esprit Sdn Bhd, located at Sg Dua, Penang, in the form of chips and flakes. The oil palm trunk and rubberwood were chipped, grinded and sieved to obtain 40 mesh fines for the further process. The fines were stored at 20 ± 2 °C with a relative humidity of 65 ± 5% prior to the pyrolysis process.

### 2.2. Pyrolysis Process

The grinded rubberwood and OPT sawdust were pyrolyzed under various stages of temperature to produce pyroligneous acid. In this study, 500 g of sawdust was weighed and loaded in a flask and was heated with a heating mantle. The sample was pyrolyzed with a heating rate of 10 °C min^−1^ from room temperature to the desired temperature in an N_2_ atmosphere. The liquid phase was collected at 300, 400 and 500 °C, respectively, by condensing the gases released from the pyrolysis process. The condenser and traps were washed with dichloromethane to dissolve oil and separate the water contained in the oil. Water was separated from the oil with a separation funnel, and the water was weighed as a pyroligneous acid product: pyroligneous acid from rubberwood (RWPA) and pyroligneous acid from oil palm trunk (OPTPA). 

### 2.3. Evaluation of Physicochemical Properties of Pyroligneous Acid

The viscosity of the pyroligneous acids was recorded using a Brookfield viscometer. The gas chromatography/mass spectroscopy (GC-MS) analysis of the pyroligneous acid was performed using the Agilent Technologies GC 6850 with a 5975 C mass selective detector (MS) and a 30 mm × 250 mm × 0.25 mm capillary column. The oven temperature was started at 50 °C for 2 min, was increased to 250 °C at a rate of 6 °C/min and was held at this temperature for 5 min. The injector port temperature and the detector temperature were set at 250 °C. The carrier gas, helium, was set at a flow rate of 1.0 mL/min. An injection volume of 1.0 µL was used. Components were identified by matching their mass spectra with those recorded in the NIST mass spectra library.

### 2.4. Vacuum Pressure Impregnation Treatment on Wood with Pyroligneous Acid

The rubberwood samples with dimensions of 25 mm in width, 25 mm in length and 7 mm in thickness (25 mm × 25 mm × 7 mm) were used in fungi and termite tests. For the mould test, the samples were cut into blocks with dimensions of 20 mm in width, 70 mm in length and 7 mm in thickness (20 mm × 70 mm × 7 mm). The rubberwood blocks were impregnated with four different concentrations of pyroligneous acid (RWPA and OPTPA) produced using a pyrolysis temperature of 500 °C (100%, 50%, 30% and 10% concentrations) and a one-liter pressure chamber. The testing variables of the vacuum pressure impregnation treatment are shown in Table 1.

The pyroligneous acid solution was impregnated into the test blocks using 130 psi for 30 min. The test blocks were then maintained in the pressure chamber for another 30 min for the final vacuum treatment schedule at 25 mmHg. The excess solution on the sample surface was wiped off and allowed to dry until a constant weight was obtained. The weight gain percentage of the pyroligneous acid solution in the treated wood was calculated according to the equation (Equation (1)) below:

Weight gain (%) = (W2 − W1)/W1 × 100(1)

where

W1 = Weight of the test block before being pressure treated with pyroligneous acid
W2 = Weight of the test block after being pressure treated with pyroligneous acid

### 2.5. Biodegradation Properties of Wood Impregnated with Pyroligneous Acid

#### 2.5.1. Determination of the Efficacy of Pyroligneous Acid Modification on Rubberwood against Moulds under Laboratory Conditions

The specimens with dimensions of 70 × 20 × 7 mm were inoculated with a spore suspension of moulds (*Penicillium* spp.), which contained about 12 × 104 spores per ml. The inoculation with spore suspensions onto the specimen was performed in sterile conditions. Absorbent papers (8–10 layers) were first placed on the bottom of each petri dish. The papers were wetted with water until free water appeared. Two glass rods were placed on top of the papers, and the treated rubberwood was placed on the glass rods. Then, 0.55 mL of the spore suspension was pipetted to the front face of the specimen, and the suspension was spread on the front face with a spatula. For each condition, five replicates were tested. The samples were then incubated for 4 weeks at 28 °C and 85% relative humidity to allow for fungi development. Fungi growth and the intensity of the development were then assessed according to the ASTM D 4445-91 [20] rating scheme, as presented in Table 2.

#### 2.5.2. Determination of the Efficacy of Pyroligneous Acid Modification on Rubberwood against Decay Fungi under Laboratory Conditions

The specimens with dimensions of 25 × 25 × 7 mm^3^ were exposed to white rot fungi, *Pycnoporus sanguineus*, based on an adaptation of the ASTM D1413-99 [21] test protocol. The specimens were added to a decay chamber containing a soil medium which was previously sterilized at 121 °C for 20 min and inoculated with the white rot fungi. The fungi were grown on sapwood feeder strips for 1 week prior to the exposure of the specimens to the decay chambers. Six decay chambers were used for each treated rubberwood, and the fungus inoculated decay chamber served as the control. The assembled decay chambers were incubated in the dark for 8 weeks (28 °C, 85% RH). After 8 weeks, the samples were cleaned and dried, and their mass loss due to fungal degradation was determined. The resistant classes of preservatives based on weight loss are shown in Table 3.

#### 2.5.3. Determination of the Efficacy of Pyroligneous Acid Modification on Rubberwood against Subterranean Termites under Laboratory Conditions

The specimens with dimensions of 25 × 25 × 7 mm were exposed to groups of subterranean termites, *Coptotermes curvignathus*, by “forced-feeding”. The four-week laboratory termite tests were based on an adaptation of the ASTM D 3345-22 [22] protocol by exposing the test blocks to the subterranean termite. The *Coptotermes curvinathus* termites were collected from an infested tree at Universiti Putra Malaysia. The specimens were individually placed in test devices (glass containers filled with sand) and exposed to subterranean termites. In these tests, rubberwood treated with pyroligneous acid was the only source of food given to termites. The containers were maintained at 25 °C and 80% RH for 4 weeks. The termite activity in each bottle was observed. At the end of the testing period, the percentage of weight loss due to termite attacks and the termites’ mortality rate were calculated. The rating scale used for the visual assessment of termite attacks is shown in Table 4, and the classification of termite mortality is shown in Table 5.

### 2.6. Statistical Analysis

Statistical analyses were carried out using the statistical package SPSS for Windows, version 16.0 (SPSS, Chicago, IL, USA). Analysis of variance (ANOVA) was used to evaluate the effect of the DMDHEU concentration and drying temperature on the physical and biological durability of the modified OPT. The effects were considered to not be statistically significant when the *p*-value was higher than 0.05 at the 95% confidence level.

## 3. Results and Discussion

### 3.1. Physicochemical Characteristics of Pyroligneous Acid

The characteristics for wood blocks impregnated with pyroligneous acid are shown in Table 6. Based on the initial dry wood mass, the pyroligneous acid yield from OPT was slightly higher than that from rubberwood. The pH values of the RWPA and OPTPA were in the range of 2.6–3.0, which is highly acidic. This is because pyroligneous acid contains high amounts of volatile acids such as acetic acid and formic acid [23] and possesses mild corrosive properties. Both pyroligneous acids had viscosities ranging from 8 to 12 cP. The viscosity of the pyroligneous acid was affected by the water content, the number of light compounds and the aging period [23]. Viscosity is a parameter that measures the resistance of a fluid to flow. Therefore, viscosity is an important factor to impregnate chemicals into wood uniformly and deeply.

Table 7 and Table 8 identify the compounds of RWPA and OPTPA. In general, a total of 24 and 23 main compounds divided into 5 groups were identified in RWPA and OPTPA, respectively. The components of the pyroligneous acids could be categorized into acids, alcohols, furfural and furan derivatives as well as phenol and methoxyphenol derivatives. The results also showed that the varieties and relative contents of those compounds in the pyroligneous acids were affected by pyrolysis temperatures. Many of the compounds were not detected from RWPA and OPTPA produced from a low pyrolysis temperature of 300 °C (17 and 16 main compounds found in RWPA and OPTPA, respectively) and were only detected at a higher pyrolysis temperature of 500 °C (23 and 22 main compounds found in RWPA and OPTPA, respectively). However, there are also a few compounds that decreased in terms of relative percentage with the increase in temperature, such as acetic acid, furfural, 5-methyl-2-furancarboxaldehyde and furfural. 5-methyl-2-furancarboxaldehyde was only detected in both types of pyroligneous acid produced at 300 °C.

Acetic acid is believed to originate from acetyl groups in the hemicellulose, which had the largest content of pyroligneous acid obtained in this study. RWPA possessed higher concentrations of acetic acid than OPTPA. Butanoic acid was only found in RWPA. Higher ketones are apparently formed from the corresponding carboxylic acids [24]. 1-Hydroxy-2-butanone and 1-(2-Furanyl) ethanone were only found in RWPA, while 2-cyclopenten-1-one was only found in OPTPA. Furfural and furane bodies are largely derived from pentosans. Only two furfural and furan derivatives were detected in small amounts: furfural and 5-methyl-2-furancarboxaldehyde.

Lignocellulosic materials such as wood are composed of mainly cellulose, hemicellulose and lignin. In this study, many phenol derivatives were found, and those in higher concentrations were phenol (4.56–4.77% in RWPA and 5.51–5.94% in OPTPA) and 2-Methoxy-phenol (2.99–3.45% in RWPA and 3.21–4.85% in OPTPA). Overall, higher phenol derivatives were found in OPTPA. 2-methoxy-5-methyl-phenol was not found in RWPA. Phenolics basically result from the thermal degradation of lignin. Lignin is a high-molecular-mass randomly cross-linked polymer consisting of an irregular array of differently bonded hydroxy- and methoxy-substituted phenylpropane units. During pyrolysis, competing thermal degradation reactions take place that generate different bond cleavages according to their bond energies, providing a high number of products due to the high structural diversity of lignin [16].

In fact, the thermal cracking of wood is the degradation of hemicelluloses, cellulose and lignin [25]. The micellar-like fragments produced by pyrolysis were degraded to smaller compounds by dehydration, dehydrogenation, deoxygenation and decarboxylation [2]. Meanwhile, the reactions of rearrangement among those smaller compounds occurred via condensation, cyclization and polymerization. In these reactions, some new compounds were produced. In this study, the content of water was the highest in the pyroligneous acids, while acetic acid was the main compound. In thermal analysis, the rates of pyrolysis of hemicelluloses and cellulose were fast. The weight loss of hemicelluloses happened at 220 to 315 °C, while that of cellulose occurred at 315 to 400 °C. However, lignin was more difficult to decompose, since its weight loss happened in a wide range of temperatures (from 160 to 900 °C) [26]. In the cracking process of the three compounds, the destructive reaction of cellulose was controlled by the decrease in the polymerization degree. However, lignin was converted into phenol, guaiacol, syringol, pyrocatechol and their derivatives. It was suggested that the aromatics and phenols and their substituted fractions of alkyl, which were the preliminary degradation products of lignin, were formed by recombination and cyclization reactions via aldol condensation from C2, C3 and C4 fragments [27]. Further reaction may produce furans, aldehyde and ketones.

Moreover, the extraction by supercritical water and the partial reactions of liquefaction occurred during the pyrolysis process. Acetic acid was formed during the thermal decomposition of hemicelluloses and lignin. In the pyrolysis reactions of biomass, water was formed by dehydration; acetic acid came from the elimination of acetyl groups originally linked to the xylose units and lignin, while furfural was formed by the dehydration of the xylose units [28].

### 3.2. Chemical Retention

**Figure 1:**
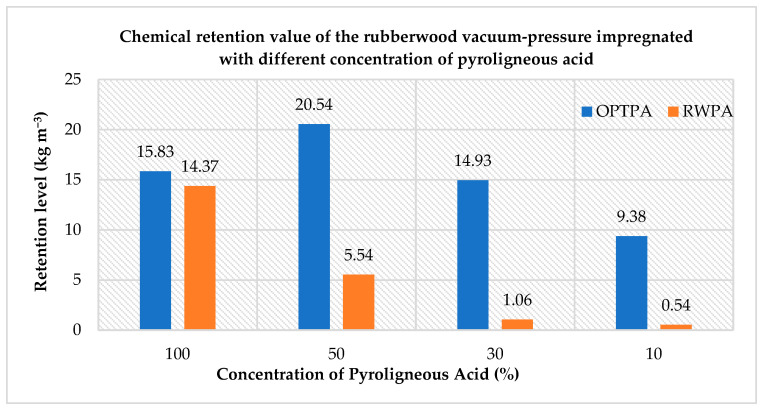
Chemical retention value of the rubberwood vacuum pressure impregnated with different concentrations of pyroligneous acid.

As shown in Figure 1, the weight gain of all the treated blocks ranged from 10.54–25.83 kgm^−3^. In most cases, OPTPA resulted in a higher retention in the wood blocks compared to RWPA. The highest retention value was obtained in the wood blocks treated with a 50% concentration of OPT pyroligneous (30.5 kgm^−3^), followed by 100% and 30% concentrations of OPT pyroligneous. RWPA resulted a lower retention value in the wood blocks.

### 3.3. Biological Durability of Rubberwood Treated with Pyroligneous Acid

#### 3.3.1. Protection against Mould

Wood blocks treated with RWPA and OPTPA were inoculated with a spore suspension of *Penicillium* sp. For each treatment condition, ten replicates were tested. The fungi growth and intensity of development were then assessed (according to Table 2). The average ratings of pyroligneous acid-treated wood blocks in terms of resistance to *Penicillium* sp. are depicted in Table 9. Normally, at moisture contents greater than 20%, mould establishment occurs on untreated rubberwood between 24 and 48 h, if temperatures permit, and rapid drying of the wood does not occur. Consequently, the mould will continue to grow and cover the wood surface.

The ratings recorded on the OPTPA-treated wood blocks on Day 28 ranged between 1 (up to 10% growth) and 5 (very heavy and tight growth) for the percentage area of fungal growth on the wood’s surface. For RWPA, the ratings ranged between 0 (no growth) and 4 (more than 50% of growth) for the percentage area of fungal growth on Day 28. The results showed that when pyroligneous acid is applied to wood blocks, the pyroligneous acid prevents mould growth to some degree and limits its intensity when compared to an untreated wood block (rating 5).

Among the treatments applied, the wood blocks impregnated with OPTPA showed better results than those treated with RWPA. The wood blocks impregnated with OPT100, OPT50 and OPT30 inhibited mould growth for the whole testing period. The wood block treated with OPT30 sufficiently inhibited mould colonization. Even with the highest concentrations of RW pyroligneous acid (RW100) applied, mould growth was observed on Day 25. Test blocks treated with RW30 and RW10 failed to provide adequate protection against mould growth, as 100% mould coverage were observed in the 4th week. On the other hand, the test blocks treated with OPT10 only slightly inhibited mould growth, with 80% of the wood surface being covered with mycelium, causing severe staining on the test block, on Day 28.

#### 3.3.2. Resistance against Decay Fungi

**Figure 2:**
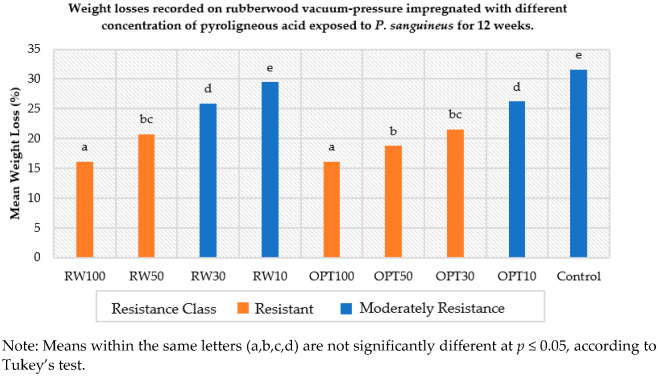
Weight losses recorded on rubberwood vacuum pressure impregnated with different concentrations of pyroligneous acid exposed to *P. sanguineus* for 12 weeks.

Resistance against decay fungi was evaluated by assessing the weight losses of the treated wood samples after 12 weeks of exposure. Ten replicates per condition were tested. The results are presented in Figure 2. The weight losses and resistance levels of test blocks treated with OPT and RW pyroligneous acid of different concentrations are shown in Figure 2. The concentrations of pyroligneous acid significantly affected the mean weight loss of the treated test blocks. The weight loss was significantly inhibited by test blocks treated with OPT100 and RW100. RW50, OPT50 and OPT30 imparted resistance in rubberwood test blocks to the white-rot fungus with weight losses of less than 25%.

The fungus *P. sanguineus* was more aggressive on the untreated sample and the test blocks treated with RWPA compared to those treated with OPTPA (OPTPA < RWPA < untreated). The two pyroligneous acid treatments lowered fungal degradation, but pyroligneous acid concentrations lower than 30% did not protect wood blocks from fungal degradation, as high mass losses were recorded (up to 29.46%, which is not different from what was observed in the untreated test blocks).

#### 3.3.3. Resistance against Subterranean Termites

The survival rate of termites, the degradation rate of wood samples (ranging from 0 = no attack to 4 = strong attack, according to the scale presented in Table 5) and the mean amount of wood consumed following the 4-week incubation in a no-choice termite test are summarized in Table 10. The termite mortality for treated wood specimens following the 4-week incubation in a no-choice termite test is summarized in Table 10. Both test blocks treated with OPTPA and RWPA pyroligneous acid, regardless on the concentrations, caused 100% termite mortality and exhibited lower weight losses (from 16 to 32%) compared to the untreated test blocks, with a low mortality at 25%. They suffered a significantly high weight loss at 44%.

Generally, the termites in all containers with treated wood blocks were active for the first week of the experiment, with elaborate tunnelling observed in all glass jars. How-ever, in the second week, the termites in the treated samples became inactive, and no activity was observed in the third week of the experiment, except for the treated samples with concentrations 30% and below. Most of the termites in containers with treated wood blocks died in the second week of the experiment. The termites in the control sample remained active throughout the whole experiment and established more elaborate tunnel systems.

The termite mortality was total (survival % = 0), with considerably high weight losses observed with test blocks treated with pyroligneous acid, suggesting the possible toxicity of pyroligneous acid when it is used as the sole source of food by termites. Pyroligneous acid provided rubberwood test blocks with little protection against the subterranean termite, *Coptotermes curvignathus*, as all samples treated with pyroligneous acid had mass losses higher than 16%. Rubberwood treated with OPTPA and RWPA is considered as slow-acting toxic bait, as most termites died in the second week after feeding on the wood block (100% termite mortality in the fourth week), and the weight loss and visual rating of the wood showed that the termites had been chewing and biting off a considerable number of fragments from the wood block (weight loss ≥16%; mean visual rating ≥3). This explains why even rubberwoods treated with the highest concentrations of pyroligneous acid create a lethal effect but are still palatable to termites under no-choice feeding.

Notably the three pyroligneous acid concentrations—OPT10, RW30 and RW10—provided much lower protection against subterranean termites, that is, mass losses at 29%, 28% and 32% compared to the mass loss of 44% of the untreated controls. Test blocks treated with higher pyroligneous acid concentrations were less degraded, with the OPT100 and RW100 samples being the less attacked ones (mean rating of 3, light attack), while the test blocks treated at a lower retention rate of 10% were heavily degraded (mainly, ratings of 1 were recorded). This observation suggests that a dose-response effect may exist.

#### 3.3.4. Discussion on the Biological Durability of Rubberwood Treated with Pyroligneous Acid

The results of the present studies demonstrate the preservative potential of RWPA and OPTPA in protecting rubberwood under laboratory conditions. In general, OPTPA has better biological durability against mould, decay fungi and termites compared to RWPA. The compounds contained in the pyroligneous acid could have played an important role in inhibiting mould growth, decay fungi and termite attacks on the treated wood.

Pyroligneous acid is comprised of water (10–20%), a mixture of carboxylic acids, several aldehydes and alcohols and pyrolytic lignin. Acetic acid is an important chemical reagent and industrial chemical. Among the organic acids, acetic acid was identified as the major compound in both RWPA and OPTPA. As both OPTPA and RWPA have almost the same relative percentage of organic acids (especially acetic acid), these components may not have a major influence on the biological durability, as there is a big difference in terms of the durability results of wood treated with RWPA and OPTPA. The antimicrobial properties of organic acids depend on the physiological status of the cells, the physicochemical properties of the surrounding environment and, in particular, the extent of the dissociation of the acid [29,30]. It was also reported that organic acids showed weak activities of growth inhibition against microorganisms [31,32,33].

Phenolic compounds are an important group identified in pyroligneous acid. Phenol and its derivatives were the primary compounds in RWPA (14.95%) and OPTPA (21.66%). Phenolic compounds such as phenol and cresols have been well known as antimicrobial agents. Phenol has some therapeutic value as a fungicide, antiseptic and disinfectant, with activity against a wide range of microorganisms, including some viruses [34]. Phenolic compounds might considerably contribute to the antimicrobial activities of the pyroligneous acids, because the total content of the phenolic compounds was presented as the main chemical components. Phenol and 2-methoxy-phenol were observed as the major phenolic compounds in both types of pyroligneous acid, with higher relative percentages in OPTPA. Therefore, phenol and 2-methoxy-phenol (guaiacol) might contribute to inhibiting mould coverage, decay fungi and termite attacks. Guaiacol present in pyroligneous acid has antioxidant properties and is used medicinally as an expectorant, antiseptic and local anaesthetic [35]. According to Langa-lomba et al. [36], guaiacol was effective against sap-staining fungi (*Ophiostoma* spp.). 4-methyl-2-methoxy-phenol (creosol) is a flavoring agent present in several foods and beverages [3]. Cresols (o, m and *p*-cresol or 2,3 and 4-methy-phenol) are used as local antiseptics, parasiticides, disinfectants and intestinal antiseptics [37]. Salim et al. [6] showed that pyroligneous acid contains guaiacol, cresol, 4-ethyl-2-methoxy-phenol and 2,6-dimethoxy-phenol and has good antimicrobial activity.

Furfural (2-furaldehyde) has numerous industrial uses; it could be used as a solvent and starting material for the production of various agrochemicals, pharmaceuticals and fragrances [38]. It is known to possess antityrosinase and antimicrobial activities against *Bacillus subtilis* and Salmonella bacteria [39]. The presence of furfural has been shown in plant extracts, and it appeared to have antifungal activity [40,41,42]. 3-methyl-2-cyclopenten-1-one is a natural compound used for food flavoring and perfumes [3,43]. It is one of the main components in maple lactone, which is commonly used in cockroach attractant traps due to its odor, which is typical of stale beer. It is not known if this chemical odor might attract termites.

The higher contents of guaiacol, phenol, cresols and furfural present in OPTPA explain its higher antifungal activities compared to RWPA. It was pointed out by Pimenta et al. [3] that the antifungal activity of pyroligneous acid from different sources cannot be attributed to a single compound but instead to a combination of several compounds. On the other hand, pyroligneous acid treatments significantly reduced the weight loss of treated rubberwood and increased termite mortality in the termite test. Test blocks treated with the highest concentrations of pyroligneous acid create a lethal effect but are still palatable to termites under no-choice feeding. In the current study, this RWPA was effective in killing the termites but had a lower protection on wood compared to OPTPA. Both types of pyroligneous acid are considered to have toxic action against termites. Higher retention levels after the impregnation treatment of rubberwood were observed using OPTPA compared to RWPA, regardless of pyroligneous acid concentrations. As the concentration of pyroligneous acid reduced, the retention level of RWPA in rubberwood decreased drastically. This might be one of the distinctive reasons for the different biological durability results of the rubberwood treated with OPTPA and RWPA besides the chemical composition.

## 4. Conclusions

The concentration of pyroligneous acid is an important factor in the effectiveness of controlling wood against biological attacks. Concentrations of 50% RWPA and 30% OPTPA were sufficient against mould growth. The same results were also observed in the effectiveness of pyroligneous acids against decay fungi using concentrations of 50% RWAPA and 30% OPTPA. However, both types of pyroligneous acids, regardless of the concentrations, showed slighter effect against termite attacks. Rubberwoods treated with OPTPA and RWPA were highly effective against mould and fungi decay; however, they are still prone to termite attacks, even with the highest concentration. OPTPA and RWPA acted as a slow-acting toxic bait to cause a high termite mortality rate due to toxic feeding, and they do not serve as good repellents to prevent termites from feeding on the wood. Still, the rubberwood treated with higher concentrations was slightly less degraded, which may suggest a dose-response effect. In general, OPTPA has better biological durability against mould, decay fungi and termites compared to RWPA. The higher retention level of OPTPA in treated rubberwood and the combination of chemical compounds in OPTPA could have been the reasons for the higher biological durability of the treated rubberwood to inhibit mould growth, decay fungi and termite attacks on the treated rubberwood. Further studies are needed to increase the retention level of pyroligneous acid in wood. Pyroligneous acids are water-soluble and are thus leached out easily when used for wood protection. The addition of a hardener to fix the chemical compounds of pyroligneous acid in wood by means of in situ polymerization has to be further investigated.

## Figures and Tables

**Table 1:** Types of pyroligneous acid and concentrations used in the vacuum pressure impregnation treatment.

Designation	Type of Pyroligneous Acid	Concentration (%)
RW100	Rubberwood	100
RW50	Rubberwood	50
RW30	Rubberwood	30
RW10	Rubberwood	10
OPT100	Oil Palm Trunk	100
OPT50	Oil Palm Trunk	50
OPT30	Oil Palm Trunk	30
OPT10	Oil Palm Trunk	10

**Table 2:** The rating scheme used to evaluate the development of mould growth on the surface of the treated wood specimens.

Rating	Percentage Area of Growth	Intensity of Visible Growth
0	No growth on the surface	Spores not activated
1	Up to 10% growth on the surface	Initial stages of growth
2	Between 10 and 30% growth on the surface	New spores produced
3	Between 30 and 50% growth on the surface	Moderate growth
4	More than 50% growth on the surface	Plenty of growth
5	Very heavy and tight growth	Coverage around 100%

Note: Based on an adaptation of the ASTM D 4445-91 [20].

**Table 3:** Resistant classes of preservatives based on weight loss.

Average Weight Loss (%)	Resistance Class
0–10	Highly resistant
11–24	Resistant
25–44	Moderately Resistant
45 and above	Slightly Resistant

Note: Based on an adaptation of ASTM D1413-99 [14].

**Table 4:** The rating scale used for the visual assessment of termite attacks was based on an adaptation of the ASTM D 3345-22 [6].

Rating	Description
4	No attack
3	Light attack: superficial erosion of insufficient depth to be measured on an unlimited area of the test specimen; or attack to a depth of 0.5 mm provided that this is restricted to an area or areas not more than 30 mm^2^ in total; or a combination of i and ii
2	Moderate attack: erosion of 1 mm in depth limited to not more than 1/10 of the surface area of the test specimen; or single tunnelling to a depth of up to 3 mm; or a combination of i and ii
1	Heavy attack: erosion of <1 mm in depth over more than 1/10 of the surface area of the test specimen; or erosion of >1 mm to <3 mm in depth limited to not more than 1/10 of the surface area of the test specimen; or isolated tunnelling of a depth >3 mm not enlarging to form cavities; or any combination of i, ii or iii
0	Failure: erosion of >1 mm to <3 mm in depth of more than 1/10 of the surface area of the test specimen, or tunnelling penetrating to a depth >3 mm and enlarging to form a cavity in the body of the test specimen, or a combination of i and ii

**Table 5:** The classification of termite mortality was based on an adaptation of the ASTM D 3345-22 [6].

Classification	Mortality Percentages
Slightly	0–33
Moderate	34–66
Heavy	67–99
Complete	100

**Table 6:** Characteristics of pyroligneous acid.

Types of Pyroligneous Acids	Temperature	Colour	pH	Viscosity (cP)	Gravimetric Yields (wt%)	
					Based on Initial Dry Wood Mass	Based on Total Pyrolysis Liquids
RWPA	300	Black brown	2.8	10	25.5	68.2
	400	Black brown	2.9	11	21.4	66.3
	500	Black brown	2.9	13	18.7	64.2
OPTPA	300	Reddish brown	2.6	8	28.2	70.4
	400	Reddish brown	2.9	10	25.7	67.1
	500	Reddish brown	3.0	12	22.8	64.5

**Table 7:** GC-MS analysis for the chemical composition of rubberwood pyroligneous acid.

Identified Peak	Compound	RWPA Relative Percentage (%)			
		RT (min)	300 °C	400 °C	500 °C
1	Cyclopentanone	4.47	0.11	0.23	0.27
2	2-Methyl-2-cyclopenten-1-one	9.69	0.38	0.54	0.83
3	1-Hydroxy-2-butanone	9.98	0.3	0.78	0.86
4	1-(2-Furanyl)-ethanone	11.56	-	0.32	0.38
5	3-Methyl-2-cyclopenten-1-one	12.93	0.14	0.44	0.61
**Total**	**Ketones**		**0.93**	**2.31**	**2.95**
6	Acetic acid	8.62	14.51	14.05	13.78
7	Propanoic acid	12.89	1.25	1.32	1.32
8	Butanoic acid	14.11	-	0.61	1.16
9	2-Methyl-propanoic acid	14.66	-	0.14	0.21
10	Tetradecanoic acid	26.11	-	0.23	0.58
**Total**	**Organic acids**		**15.76**	**16.35**	**17.05**
11	Furfural	8.16	0.62	0.45	0.41
12	5-Methyl-2-furancarboxaldehyde	12.31	0.16	-	-
**Total**	**Aldehydes**		**0.78**	**0.45**	**0.41**
13	Methyl-4-oxo-pentanate	12.76	0.27	0.21	0.18
14	Methyl 4-hydroxybutanoate	19.15	-	-	0.31
**Total**	**Esters**		**0.27**	**0.21**	**0.49**
15	Phenol	8.11	4.77	4.67	4.56
16	2-Methoxy-phenol (guaiacol)	12.65	2.99	3.07	3.45
17	2-Methoxy-5-methyl-phenol	-	-	0.19	0.32
18	2-Methoxy-4-methyl-phenol	16.72	1.18	1.37	1.67
19	4-methyl-2-methoxyl-phenol (creasol)	18.17	2.99	2.45	2.38
20	4-Methyl-phenol (ρ-creasol)	19.57	0.41	0.52	0.56
21	2-Methoxy-4-propyl-phenol	20.62	0.21	0.21	0.39
22	3-Methoxy-1,2-benzenediol	20.71	1.38	1.72	1.98
23	3-Methoxy-5-methyl-phenol	21.21	-	1.51	2.16
24	1,2-Benzenediol	22.11	-	0.28	0.84
**Total**	**Phenol and derivatives**		**13.93**	**15.99**	**18.31**

**Table 8:** GC-MS analysis for the chemical composition of oil palm trunk pyroligneous acid.

Identified Peak	Compound	OPTPA Relative Percentage (%)			
		RT (min)	300 °C	400 °C	500 °C
1	Cyclopentanone	5.47	-	0.18	0.21
2	2-Cyclopenten-1-one	8.78	0.67	0.66	0.78
3	2-Methyl-2-cyclopenten-1-one	9.19	0.36	0.54	0.64
4	1-Hydroxy-2-butanone	9.47	0.33	0.41	0.47
5	3-Methyl-2-cyclopenten-1-one	12.16	0.45	0.65	0.76
**Total**	**Ketones**		**1.81**	**2.26**	**2.65**
6	Acetic acid	11.12	14.25	13.34	13.21
7	Propanoic acid	12.89	0.57	1.29	1.31
8	2-Methyl-propanoic acid	14.66	-	0.23	0.21
9	Tetradecanoic acid	30.11	-	0.18	0.98
**Total**	**Organic acids**		**14.82**	**15.04**	**15.71**
10	Furfural	10.16	0.72	0.55	0.45
11	5-Methyl-2-furancarboxaldehyde	15.31	0.16	-	-
**Total**	**Aldehydes**		**0.88**	**0.55**	**0.45**
12	Methyl-4-oxo-pentanate	13.77	-	0.31	0.23
13	Methyl 4-hydroxybutanoate	19.65	-	-	0.21
**Total**	**Esters**		**0**	**0.31**	**0.44**
14	Phenol	9.14	5.94	5.63	5.51
15	2-Methoxy-phenol (guaiacol)	14.65	3.21	4.15	4.85
16	2-Methoxy-5-methyl-phenol	15.89	-	0.23	0.37
17	2-Methoxy-4-methyl-phenol	17.72	1.15	1.21	1.29
18	4-methyl-2-methoxyl-phenol (creasol)	18.57	2.3	2.42	2.50
19	4-Methyl-phenol (ρ-creasol)	19.77	0.45	0.66	0.68
20	2-Methoxy-4-propyl-phenol	20.02	0.22	0.21	0.29
21	3-Methoxy-1,2-benzenediol	20.59	1.78	1.92	1.98
22	3-Methoxy-5-methyl-phenol	21.18	-	-	2.56
23	1,2-Benzenediol	22.15	-	-	1.51
**Total**	**Phenol and derivatives**		**15.05**	**16.43**	**21.54**

**Table 9:** Mould resistance ratings of the rubberwood test block vacuum pressure impregnated with different concentrations of pyroligneous acid.

Treatments	Appearance of Mould (Day)	Average Rating on Mold Growth			
		Week 1	Week 2	Week 3	Week 4
RW100	25	0	0	0	1
RW50	22	0	0	1	3
RW30	9	0	1	3	5
RW10	3	2	3	4	5
OPT100	no mould growth	0	0	0	0
OPT50	no mould growth	0	0	0	0
OPT30	no mould growth	0	0	0	0
OPT10	4	1	2	3	4
Untreated	2	2	3	4	5

**Table 10:** Mean of termite survival rates, quantity of wood consumed and degradation ratings recorded at the end of the tests on the test blocks treated with pyroligneous acid.

Treatments	Termite Mortality		Mean Weight Loss (%)	Rating on Visual Assessment of Termite Attack (Nmber of Samples per Rating)				
	%	Classification		0	1	2	3	4
RW100	100 ^a^	Complete	17.09 ^ab^			1	4	
RW50	100 ^a^	Complete	20.72 ^bc^			3	2	
RW30	100 ^a^	Complete	27.88 ^c^		4	1		
RW10	100 ^a^	Complete	32.46 ^e^	1	4			
OPT100	100 ^a^	Complete	16.12 ^a^				5	
OPT50	100 ^a^	Complete	18.78 ^b^			4	1	
OPT30	100 ^a^	Complete	22.55 ^bc^		3	2		
OPT10	100 ^a^	Complete	29.23 ^d^		5			
Untreated	25.4 ^b^	Slightly	43.58 ^f^	3	2			
Pr > F	**		**					

Note: Means followed by the same letters (a,b,c,d) in the same column are not significantly different at *p* ≤ 0.05 according to Tukey’s test. n.s.: not significant at *p* > 0.05; **: significant at *p* ≤ 0.05.

## Data Availability

All relevant data are within the manuscript.
